# Protein-rich food intake frequency score and muscle mass, strength, muscle-specific strength or physical performance in Japanese older women: a cross-sectional study

**DOI:** 10.1186/s12877-025-06932-3

**Published:** 2026-01-13

**Authors:** Yui Nakayama, Keiichi Yokoyama, Ai Moriyasu, Mika Kimura, Tsukasa Yoshida, Hisamine Kobayashi, Misaka Kimura, Yosuke Yamada

**Affiliations:** 1 Department of Physical Activity Research, National Institute of Health and Nutrition, Health and Nutrition, National Institutes of Biomedical Innovation, Osaka, 566-0002 Japan; 2 Center for Health Promotion, International Life Sciences Institute, Tokyo, 135-0004 Japan; 3 Institute of for Active Health, Kyoto University of Advanced Science, Kyoto, 621-0022 Japan; 4 Ajinomoto Co, Inc, Tokyo, 104-8315 Japan

**Keywords:** sarcopenia, nutrition, grip strength, knee extension strength, chair stand, appendicular skeletal muscle mass

## Abstract

**Background:**

Sarcopenia, defined as age-related muscle loss, has significant implications on the physical performance and health of older adults. An adequate dietary protein intake plays a crucial role in maintaining muscle mass and function. In Japan, the “Take 10!” assessment method, focusing on 10 food groups with an emphasis on 5 protein-rich foods, has been used for older adults. This study aimed to explore the relationship between the protein-rich food intake frequency score (PFFS) and muscle mass, strength, muscle-specific strength, and physical performance in older Japanese women.

**Methods:**

This study included 309 Japanese women aged 65–92 years. The appendicular skeletal muscle mass and skeletal muscle index were assessed. Hand grip strength and knee extension strength were measured, and muscle-specific strength was calculated. Chair stand test, shuttle stamina walk test, 10-m walk test, and timed up-and-go test were conducted. The Take 10 food frequency score (Take10-FFS) and PFFS were obtained. A one-way analysis of covariance was conducted, adjusted for age, percent body fat, exercise habits, smoking habits, alcohol habits, preexisting conditions, polypharmacy, fall incidence within 1 year, subjective economic status, and years of education.

**Results and conclusion:**

Higher Take 10-FFS and PFFS were associated with better muscle mass, strength, and physical performance (*P* < 0.05), emphasizing the importance of protein intake in preventing sarcopenia. However, no direct association was found between PFFS and muscle-specific strength (*P* > 0.05). This underscores the complexity of the factors influencing muscle-specific strength.

## Introduction

Sarcopenia was originally defined by Rosenberg in 1989 as an age-related loss of skeletal muscle mass [1]. In 2010, the European Working Group on Sarcopenia in Older People (EWGSOP) defined sarcopenia as the presence of low muscle mass associated with the loss of strength and/or low physical performance [2]. The definition was updated by the same group (EWGSOP2) in 2018 [3], stating that the isolated loss of strength indicates “probable sarcopenia,” and the diagnosis of sarcopenia is confirmed when the condition is accompanied by low muscle mass or muscle quality. According to the EWGSOP2 consensus, physical performance is now considered a marker of disease severity rather than a diagnostic criterion. In 2024, the Global Leadership Initiative in Sarcopenia proposed the first global conceptual definition of sarcopenia, identifying muscle mass, muscle strength, and muscle-specific strength as components of sarcopenia, with impaired physical performance as an outcome [4, 5].

Adequate dietary protein intake is essential for maintaining muscle mass and function in older adults, and enhancing dietary habits is important for preventing sarcopenia, promoting health, and improving public health [6–9]. One recommended and easily implementable method is increasing awareness of dietary variety in daily life [10–14]. Some countries incorporate dietary varieties in their dietary guidelines that are tailored to their local dietary culture [15, 16].

In Japan, the “Take 10!” assessment method is frequently used in older adults [17–19]. This method is used to assess dietary variety across 10 food groups, including 5 high protein-rich foods (meat, fish/shellfish, eggs, milk/dairy products, and soybeans/soy products), to ensure a balanced diet [12]. Previous studies demonstrated that the lower dietary variety assessed by Take 10! is associated with malnutrition and frailty in Japanese older adults [10, 11]. In addition, A previous study indicated that the protein-rich food intake frequency score (PFFS) obtained from the “Take 10!” assessment is correlated with physical performance [12]. However, no study has examined the relationship between PFFS and muscle mass, such as appendicular lean mass (ALM), skeletal muscle index (SMI), or muscle-specific strength (e.g., muscle strength/muscle size).

Dietary protein intake is necessary for muscle protein synthesis but does not appear to be directly associated with improvement in motor skills [20]. Therefore, we hypothesized that the PFFS is associated with ALM, SMI, muscle strength, and physical performance, but not with muscle-specific strength in older adults. A previous study found that the frequency of protein-rich food intakes is associated with frailty more strongly in older women than in men [7]. Thus, this study aimed to examine the relationship between PFFS or other food frequency scores and muscle mass, strength, muscle-specific strength, and physical performance in Japanese community-dwelling older women.

## Materials and methods

This was a cross-sectional study following the STROBE checklist for observational study. In this study, we conducted analyses of the women who participated in a 1-day routine physical function test for the older adults in 2019. The physical function test has been conducted annually at Kyoto University of Advanced Science (Kyoto, Japan). The participants were all community-dwelling and were invited to attend by mail. A total of 309 Japanese women aged 65–92 years were included in this study. Data on health status, habitual physical activity, and social and dietary habits were collected using a questionnaire. The study was approved by the Ethics Committee of the Kyoto University of Advanced Science (KUAS-19-5). Written informed consent was obtained from all participants. The staff who carried out the data collection previous trained.

The inclusion criteria, previously described [21], were as follows: older women who (a) reported the ability to walk without a cane, (b) had no history of lower limb trauma or surgery, (c) had no neuromuscular disorder, (d) were not taking any medication for edema or not using an artificial pacemaker, (e) had no acute disease that causes muscle weakness, (f) had no definitive kidney or digestive disorder, and (g) had the ability to provide informed consent without severe dementia.

Barefoot standing height (Ht) was measured to the nearest 0.1 cm using a stadiometer (DST-210 S, MURATEC-KDS Co., Kyoto, Japan). Body mass was measured to the nearest 0.1 kg by MC-780 (TANITA, Tokyo, Japan), with the participant dressed in light clothing without shoes.

### Appendicular lean mass and skeletal muscle mass index

A standing-posture 8-electrode, segmental, multifrequency bioelectrical impedance analysis (MC-780, TANITA, Tokyo, Japan) was used to measure the bioelectrical impedance at frequencies of 5, 50, and 250 kHz (Z_5_, Z_50_, and Z_250_, respectively) [22]. The ALM of 756 adults aged 18–86 years was calculated using a previously validated and cross-validated Eq. [22]:

$$\begin{aligned}\mathrm{Men}:\;\mathrm{ALM}=&\left(0.6947\times\left(\mathrm{Ht}^2/{\mathrm Z}_{50}\right)\right)\\&+\left(-55.24\times\left({\mathrm Z}_{250}/{\mathrm Z}_5\right)\right)\\&+\left(-10,940\times\left(1/{\mathrm Z}_{50}\right)\right)\\&+51.33\end{aligned}$$

$$\left(\mathrm R^2=0.851,\;\mathrm{SEE}=1.46\mathrm{kg}\right)$$

$$\begin{aligned}\mathrm{Women}:\;\mathrm{ALM}=&\left(0.6144\times\left(\mathrm{Ht}^2/{\mathrm Z}_{50}\right)\right)\\&+\left(-36.61\times\left({\mathrm Z}_{250}/{\mathrm Z}_5\right)\right)\\&+\left(-9,332\times\left(1/{\mathrm Z}_{50}\right)\right)\\&+37.91\end{aligned}$$

$$\left(\mathrm R^2=0.757,\;\mathrm{SEE}=1.22\mathrm{kg}\right)$$

ALM (kg) was normalized to the squared Ht [ALM/Ht^2^] and referred to as the skeletal muscle index (SMI, kg/m^2^). Upper limb muscle mass (UMM) and lower limb muscle mass (LMM) were also obtained using the MC-780 device. UMM is the total amount of muscle mass of the right and left arms, and LMM is the total amount of muscle mass of the right and left legs.

#### Muscle strength and muscle-specific strength

Hand grip strength (HGS) and isometric knee extension strength (KES) tests were conducted after obtaining bioimpedance analysis measurements [23, 24]. The maximal HGS was measured using a Smedley hand dynamometer (Grip-D, TKK5401; Takei Scientific Instruments, Niigata, Japan), as described elsewhere [25]. The participants were instructed to maintain a standard bipedal position throughout the test. The involved arm was fully extended with the dynamometer not touching any other part of the body except the hand being measured. The width of the handle was adjusted to ensure that the second phalanx was against the inner stirrup while the participant held the dynamometer. Each hand was measured alternately, with a brief rest between trials, and the highest value was recorded. The participants were encouraged to exert maximal effort during each trial. The sum of the maximum HGS recordings for each hand was used to calculate the mean [23, 24].

Maximal KES at a knee angle of 90° was measured in a sitting position on a custom-made dynamometer chair, as described previously (Yamada JAP, 2013). The ankle was attached to a strain gauge system (TKK5710e; Takei Scientific Instruments). After familiarization with the test, the participants were encouraged to produce maximal knee extension force. Each participant performed two maximal efforts, with a 1-minute rest period between attempts, and the highest value was recorded [23, 24].

The muscle-specific strength indices were calculated as follows: HGS divided by UMM (HGS/UMM) and KES divided by LMM (KES/LMM).

#### Physical performance

Chair stand test (frequency/30 s): During the test, the participant was asked to stand up from and sit down as quickly as possible on a firm, padded, 0.43-m high armless chair. The back of the chair was supported by a wall. The participants were instructed to fold their arms across their chest during the test. The number of repetitions within 30 s was recorded [23].

Shuttle stamina walk test (meters): This test was performed to assess the distance of fast walking around two poles placed 10 m apart within 3 min. The participants were instructed to [1] walk as fast as possible and [2] walk around the pole in small steps at each turn. This test required participants to exert maximal effort [23, 26].

Ten-meter Walk test (s): The participants were instructed to walk 10 m at their usual comfortable pace to assess habitual gait velocity. Subsequently, they were instructed to walk as fast as possible. The walking time was measured using a digital stopwatch [27].

Timed up and go (TUG) test (seconds): The TUG is used to evaluate the function and mobility of the lower extremities (Podsiadlo and Richardson, 1991). The participants sat on a standard chair (Ht: 0.4 m) without armrests. The time required for the participants to stand up, walk 3 m, turn around, walk back, and sit down as quickly and safely as possible was measured. The measurements were recorded using a stopwatch [28, 29].

#### Sarcopenia criteria

Since no cutoff values for sarcopenia diagnostic criteria by GLIS have been proposed at this time [4, 5], the prevalence of sarcopenia was determined based on the AWGS2019 criteria [30].

#### Frequency of food intake

**Fig. 1:**
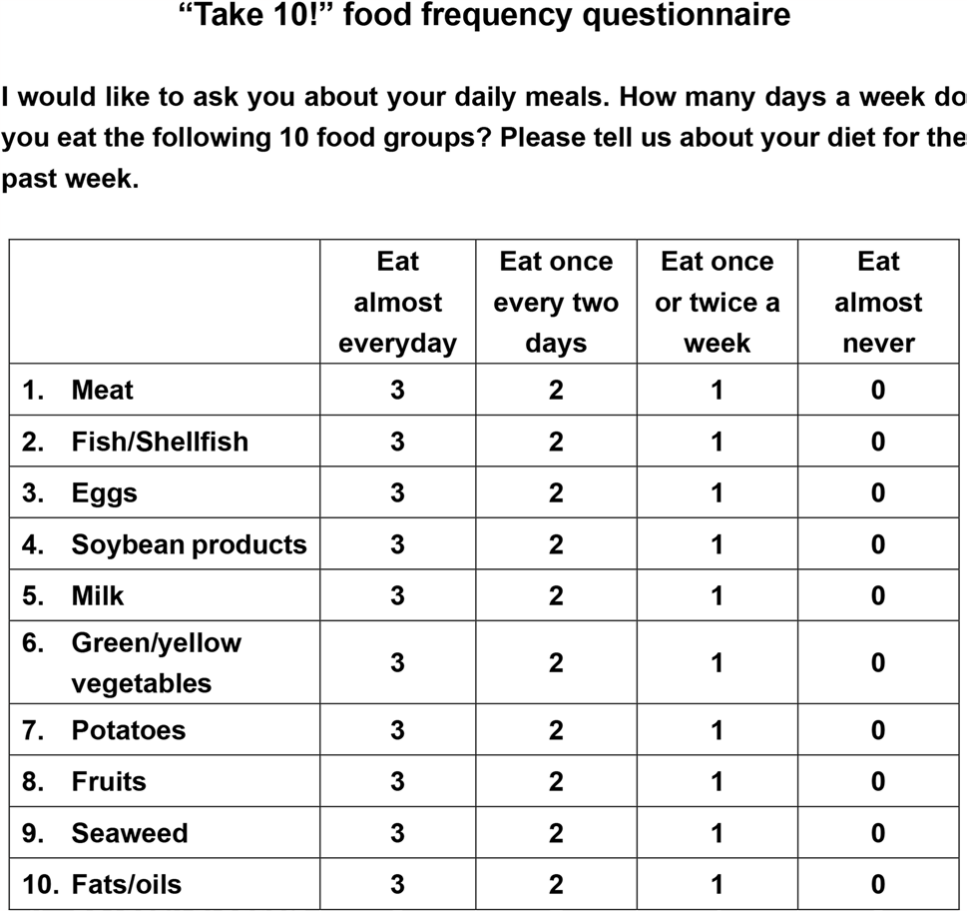
“Take 10!” food frequency questionnaire (Translated from Japanese version by authors). The “Take 10!” assessment method is frequently used for older adults in Japan [17–19]

The intake frequency of 10 food groups, such as meat, fish and shellfish, eggs, milk and dairy products, soybean products, green and yellow vegetables, potatoes, fruits, seaweed, and fats and oils, were assessed using the “Take 10!” food frequency questionnaire which had been previously developed in Japanese [17, 18] (Fig. 1). The intake frequency of each food group was determined using four response options: [1] eat almost every day (3 points) [2], eat 3 or 4 days a week (2 points) [3], eat 1 or 2 days a week (1 point), and [4] hardly ever eat (0 points). The “Take 10!” food frequency score (Take10-FFS), which evaluates dietary variety, was calculated by summing the scores for each of the 10 food groups (range: 0–30) [10, 19]. The PFFS was calculated as the sum of the scores for meat, fish, shellfish, eggs, milk, dairy products, and soybean products (range: 0–15) [12]. A score obtained by subtracting the PFFS from the Take10-FFS was calculated to exclude the PFFS data (herein referred to as ex-PFFS, range: 0–15) [12]. The participants were then categorized into tertiles based on their Take10-FFS (T1: 0–19, T2: 20–23, and T3: 24–30), PFFS (T1: 0–10, T2: 11–12, and T3: 13–15), and ex‐PFFS (T1: 0–9, T2: 10–11, and T3: 12–15)(fig. 1).

#### Other variables

Exercise habits, smoking habits, alcohol habits, preexisting conditions, polypharmacy, fall incidence within 1 year, subjective economic status, and years of education were assessed using a self-administered questionnaire.

#### Statistical analysis

All analyses were performed using IBM SPSS Statistics for Windows ver. 28 (IBM Corp., Armonk, NY, USA). We have tested the normality of the outcome using Kolmogorov-Smirnov test, and the outcome values were normally distributed. A one-way analysis of variance and trend analysis was performed to compare the physical and demographic characteristics of the participants according to the tertiles of Take10-FFS, PFFS, or ex-PFFS, and the results were expressed as the mean and standard deviation (SD). One-way analysis of covariance and trend analysis were performed to compare muscle mass, muscle strength, muscle-specific strength, and physical performance according to the tertiles of Take10-FFS, PFFS, or ex-PFFS, adjusting for age, percent body fat, exercise habits, smoking habits, alcohol habits, preexisting conditions, polypharmacy, fall incidence within 1 year, subjective economic state, and years of education. The adjusted mean was presented along with the standard error. A P value of < 0.05 was considered significant.

## Results

The physical and demographic characteristics of the participants according to the Take10-FFS, PFFS, and ex-PFFS tertiles are summarized in Table 1. The mean and SD of age, Ht, weight, and BMI were 75.3 ± 5.2 years old, 151.9 ± 5.2 cm, 49.2 ± 6.7 kg, and 21.3 ± 2.6 kg/m^2^. Significant differences were observed in Ht and/or weight, subjective economic status, and years of education between the groups. Participants with higher Take10-FFS, PFFS, and ex-PFFS had better subjective economic status, while those with higher Take10-FFS and PFFS had more years of education.

**Table 1:** Physical and demographic characteristics of the participants according to the “Take 10!” food frequency score

Take10-FFS	T1 (*n* = 93)	T2 (*n* = 121)	T3 (*n* = 95)	*P* for trend
	≤ 19	20 to 23	≥ 24	
Age (years old)	74.6 ± 5.4	75.1 ± 5.0	75.5 ± 5.1	0.533
Height (cm)	151.0 ± 5.6	151.7 ± 4.6	152.9 ± 5.4	0.015
Weight (kg)	47.8 ± 6.7	49.2 ± 6.3	50.3 ± 6.8	0.010
BMI (kg/m2)	20.9 ± 2.5	21.4 ± 2.5	21.5 ± 2.6	−0.130
Percent body fat (%)	27.7 ± 6.8	28.1 ± 6.5	28.7 ± 6.5	0.314
Subjective economic state (1 to 4)	2.64 ± 0.7	2.90 ± 0.6	2.92 ± 0.6	0.004
Years of education (years)	11.7 ± 2.3	12.4 ± 2.3	12.8 ± 2.5	0.004
				*P* values
Exercise habits (n and %)	64 (68.8%)	103 (85.1%)	71 (74.7%)	0.016
Smoking habits (n and %)	0 (0%)	0 (0%)	0 (0%)	NA
Alcohol habits (n and %)	32 (34.4%)	45 (37.2%)	36 (37.9%)	0.870
Preexisting conditions (n and %)	41 (44.1%)	49 (40.5%)	42 (44.2%)	0.818
Polypharmacy (n and %)	12 (12.9%)	7 (5.8%)	8 (8.4%)	0.187
Fall of incidence within 1 year (n and %)	17 (18.3%)	25 (20.7%)	19 (20.0%)	0.908

Values are expressed as the mean ± standard deviation. P for trends were obtained using analysis of variance. P values were obtained using chi-square tests

*BMI* body mass index, Take10-FFS, the “Take 10!” food frequency score

Table 2 shows the adjusted means for muscle mass, strength, specific strength, and physical performance according to the Take10-FFS tertiles. Take10-FFS was significantly associated with ALM, SMI, KES, chair stand, shuttle stamina walk, maximal walk, and TUG (P for trend < 0.05) but not with HGS, HGS/UMM, KES/LMM, and usual walking.

**Table 2:** Adjusted means for muscle mass, muscle strength, muscle-specific strength, and physical performance according to the “Take 10!” food frequency score tertiles

Take10-FFS	T1 (*n* = 93)	T2 (*n* = 121)	T3 (*n* = 95)	*P* for trend	
	≤ 19	20 to 23	≥ 24		
Muscle mass					
ALM (kg)	14.31 ± 0.19	14.84 ± 0.16	15.15 ± 0.18	**0.002**	T1 < T3
SMI (kg/m2)	6.246 ± 0.05	6.432 ± 0.04	6.456 ± 0.05	**0.005**	T1 < T3
Muscle strength					
HGS (kg)	21.50 ± 0.38	22.24 ± 0.33	22.31 ± 0.37	0.139	ns
KES (kg)	23.16 ± 0.74	24.55 ± 0.64	25.71 ± 0.72	**0.016**	T1 < T3
Muscle-specific strength					
HGS/UMM	14.90 ± 0.24	14.72 ± 0.20	14.76 ± 0.23	0.667	ns
KES/LMM	4.067 ± 0.12	4.128 ± 0.10	4.246 ± 0.12	0.292	ns
Physical performance					
Chair Stand (n/30s)	24.99 ± 0.61	26.10 ± 0.53	26.98 ± 0.60	**0.024**	T1 < T3
Shuttle stamina walk (m/3min)	255.6 ± 2.93	260.2 ± 2.53	267.4 ± 2.85	**0.005**	T1 < T3
Usual walk (s/10m)	7.139 ± 0.10	7.038 ± 0.08	6.867 ± 0.09	0.058	ns
Maximal walk (s/10m)	5.654 ± 0.08	5.442 ± 0.07	5.323 ± 0.08	**0.005**	T1 > T3
TUG (s)	6.982 ± 0.10	6.737 ± 0.09	6.570 ± 0.10	**0.005**	T1 > T3

Values are expressed as the mean ± standard error. P for trends were obtained using analysis of covariance. Adjusted for age, percent body fat, exercise habits, smoking habits, alcohol habits, preexisting conditions, polypharmacy, fall incidence within 1 year, subjective economic status, and years of education

*ALM* appendicular lean mass, *SMI* skeletal muscle index, *HGS* hand grip strength, *KES* knee extension strength, *TUG* timed up and go

Table 3 shows the adjusted means for muscle mass, strength, specific strength, and physical performance according to the PFFS tertiles. PFFS was significantly associated with ALM, SMI, HGS, KES, chair stand, shuttle stamina walk, usual walk, maximal walk, and TUG (P for trend < 0.05) but not with HGS/UMM and KES/LMM.

**Table 3:** Adjusted means for muscle mass, muscle strength, muscle-specific strength, and physical performance according to the protein-rich food frequency score tertiles

PFFS	T1 (*n* = 119)	T2 (*n* = 104)	T3 (*n* = 86)	*P* for trend	
	≤ 10	11 to 12	≥ 13		
Muscle mass					
ALM (kg)	14.35 ± 0.16	15.04 ± 0.17	15.04 ± 0.19	**0.009**	T1 < T3
SMI (kg/m2)	6.287 ± 0.04	6.449 ± 0.04	6.437 ± 0.05	**0.042**	T1 < T3
Muscle strength					
HGS (kg)	21.41 ± 0.33	22.38 ± 0.35	22.49 ± 0.4	**0.042**	T1 < T3
KES (kg)	22.53 ± 0.63	26.23 ± 0.67	25.09 ± 0.75	**0.011**	T1 < T3
Muscle-specific strength					
HGS/UMM	14.74 ± 0.21	14.74 ± 0.22	14.91 ± 0.24	0.583	ns
KES/LMM	3.930 ± 0.10	4.355 ± 0.11	4.192 ± 0.12	0.110	ns
Physical performance					
Chair Stand (n/30s)	25.04 ± 0.54	26.58 ± 0.57	26.75 ± 0.64	**0.045**	T1 < T3
Shuttle stamina walk (m/3min)	253.5 ± 2.53	266.1 ± 2.69	265.4 ± 3.01	**0.003**	T1 < T3
Usual walk (s/10m)	7.245 ± 0.08	6.891 ± 0.09	6.850 ± 0.10	**0.004**	T1 > T3
Maximal walk (s/10m)	5.725 ± 0.07	5.322 ± 0.07	5.294 ± 0.08	**< 0.001**	T1 > T3
TUG (s)	7.010 ± 0.09	6.624 ± 0.09	6.577 ± 0.10	**0.003**	T1 > T3

Values are expressed as the mean ± standard error. P for trends were obtained using analysis of covariance. Adjusted for age, percent body fat, exercise habits, smoking habits, alcohol habits, preexisting conditions, polypharmacy, fall incidence within 1 year, subjective economic status, and years of education

*ALM* appendicular lean mass, *SMI* skeletal muscle index, *HGS* hand grip strength, *KES* knee extension strength, *UMM* upper limb muscle mass, *LMM* lower limb muscle mass, *TUG* timed up and go

Table 4 shows the adjusted means for muscle mass, strength, specific strength, and physical performance according to the ex-PFFS tertiles. Ex-PFFS was significantly associated with ALM and SMI (P for trend < 0.05) but not with other variables.

**Table 4:** Adjusted means for muscle mass, muscle strength, muscle-specific strength, and physical performance according to the excluded protein-rich food frequency score tertiles

ex-PFFS	T1 (*n* = 100)	T2 (*n* = 107)	T3 (*n* = 102)	*P* for trend	
	≤ 9	10 to 11	≥ 12		
Muscle mass					
ALM (kg)	14.55 ± 0.18	14.59 ± 0.17	15.19 ± 0.17	**0.012**	T1 < T3
SMI (kg/m2)	6.331 ± 0.05	6.334 ± 0.04	6.486 ± 0.05	**0.031**	T1 < T3
Muscle strength					
HGS (kg)	22.00 ± 0.37	22.03 ± 0.35	22.07 ± 0.36	0.896	ns
KES (kg)	23.92 ± 0.71	24.38 ± 0.68	25.16 ± 0.69	0.216	ns
Muscle-specific strength					
HGS/UMM	15.00 ± 0.22	14.85 ± 0.22	14.51 ± 0.22	0.118	ns
KES/LMM	4.126 ± 0.12	4.176 ± 0.11	4.134 ± 0.11	0.960	ns
Physical performance					
Chair Stand (n/30s)	25.39 ± 0.59	26.92 ± 0.57	25.75 ± 0.58	0.664	ns
Shuttle stamina walk (m/3min)	256.1 ± 2.82	264.4 ± 2.72	262.4 ± 2.75	0.110	ns
Usual walk (s/10m)	7.102 ± 0.09	6.959 ± 0.09	6.991 ± 0.09	0.418	ns
Maximal walk (s/10m)	5.643 ± 0.07	5.325 ± 0.07	5.449 ± 0.07	0.079	ns
TUG (s)	6.942 ± 0.1	6.645 ± 0.09	6.701 ± 0.09	0.087	ns

Values are expressed as the mean ± standard error. P for trends were obtained using analysis of covariance. Adjusted for age, percent body fat, exercise habits, smoking habits, alcohol habits, preexisting conditions, polypharmacy, fall incidence within 1 year, subjective economic status, and years of education.

*ALM* appendicular lean mass, *SMI* skeletal muscle index, *HGS* hand grip strength, *KES* knee extension strength, *TUG* timed up and go

Only 2.9% (9 of 309 participants) of the current population developed sarcopenia based on the AWGS2019 criteria. Therefore, we could not compare the differences in Take10-FFS, PFFS, and ex-PFFS between patients with and without sarcopenia.

## Discussion

The present study investigated the association between Take10-FFS and muscle mass, muscle strength, muscle-specific strength, and physical performance in Japanese community-dwelling older women. Take10-FFS is associated with muscle mass, muscle strength, and physical performance in community-dwelling older women. This association was mostly attributed to the five-item PFFS. Meanwhile, Take10-FFS, PFFS, and ex-PFFS were not significantly associated with muscle-specific muscle strength, suggesting that while protein intake frequency may contribute to muscle preservation and function, it does not necessarily enhance the efficiency of muscle strength relative to muscle mass. The study hypotheses were confirmed by the current cross-sectional analysis.

Our results align with previous studies highlighting the importance of dietary protein intake in maintaining muscle health in older adults [6–9]. The positive relationship between PFFS and both appendicular lean mass (ALM) and skeletal muscle index (SMI) suggests that frequent intake of protein-rich foods supports muscle preservation. This is consistent with the well-established role of protein in muscle protein synthesis and in preventing age-related muscle loss [6–9]. Additionally, our findings reinforce the role of dietary variety, as measured by the Take10-FFS, in promoting overall physical health and reducing frailty risk [10, 11].

The observed association between PFFS and muscle strength (hand grip strength and knee extension strength) supports the hypothesis that dietary protein intake contributes to muscle function. This finding is particularly relevant given that muscle strength is a key determinant of mobility and independence in older adults [3]. The significant relationships between PFFS and multiple physical performance tests, including the chair stand test, shuttle stamina walk, 10-m walk test, and timed up-and-go (TUG) test, highlight the functional benefits of frequent protein intake. These findings align with previous reports indicating that higher dietary protein consumption is associated with better physical performance in older populations [12].

Interestingly, no significant association was found between Take10-FFS, PFFS, or ex-PFFS and muscle-specific strength (HGS/UMM and KES/LMM). This suggests that while protein intake supports absolute muscle strength, it does not necessarily enhance strength relative to muscle size. This finding is consistent with previous studies that indicate that protein intake alone may not directly influence neuromuscular efficiency or motor unit recruitment [20]. Given the complexity of muscle function, other factors such as resistance training, neuromuscular adaptation, and hormonal regulation likely play a role in improving muscle-specific strength [31, 32]. In addition, several interventional studies combining exercise training and nutrient intake (protein plus other nutrients) have reported that exercise training with nutrient intake is effective in improving muscle quality and quantity [33, 34]. Future studies should explore the interaction between dietary protein intake and physical activity interventions to determine whether combined approaches enhance muscle efficiency.

Ex-PFFS was associated with muscle mass alone, but not with muscle strength or physical performance. Many previous studies have reported a link between muscle mass and protein or vitamin D levels; however, some studies have noted an association between other nutrients (such as vitamins B1 and B12 [35], polyunsaturated fatty acids [36], calcium, and selenium [37]) and muscle mass. In addition, many nutrients other than proteins are involved in muscle synthesis, potentially influencing the ex-PFFS.

The participants with higher Take10-FFS, PFFS, and ex-PFFS had better subjective economic status and/or more years of education. Economic affordability is thought to influence consumers’ willingness to purchase and choose food products. In this study, an association was observed between subjective economic status and the FFS, PFFS, and ex-PFFS. The recommendation to consume these 10 food groups is straightforward, given their popularity in Japan. Local programs promoting this dietary approach have been implemented by governments and organizations in Japan [17, 18, 38]. However, further studies are warranted to explore the differences in health promotion effects by subjective economic status or years of education among the target population.

This study showed an association between PFFS and muscle mass and strength, components of sarcopenia, and physical performance, an outcome of sarcopenia. Improving the PFFS may prevent sarcopenia. By contrast, relying solely on high protein food intake may not effectively improve muscle-specific strength. Hence, an appropriate exercise program may be required.

Some limitations should be acknowledged. One limitation is that the current study did not directly measure the factors reflecting muscular-nervous system. The intake of each nutrient (such as vitamins D, B1 and B12, polyunsaturated fatty acids, calcium, and selenium) was not evaluated in the current study, and amino acid and vitamin D levels at the blood level were not measured. This is another limitation of the current study. In addition, the BIA method is not a gold standard for body composition assessment, and the measurement timing of BIA and/or standing-posture BIA device used in older people with calloused epidermis may influence the results bias. As another limitation, weight and body composition measurement times varied during the day, so there is a possibility of measurement errors on weight and body composition. The current study is a cross-sectional study; thus, cause-and-effect logic is not clear. Hence, longitudinal and intervention studies are required.

In conclusion, Take10-FFS was associated with muscle mass, muscle strength, and physical performance, but not with muscle-specific strength. These associations were mostly attributed to the five-item PFFS, but not to other food intake frequency scores. These results suggest that improving PFFS may help maintain muscle mass, strength, and/or physical performance. The “Take 10!” food frequency questionnaire is an easy and useful tool for assessing the frequency of protein-rich food intake in clinical settings.

## Data Availability

The data that support the findings of this study are not openly available due to reasons of sensitivity and are available from the corresponding author upon reasonable request.

## Abbreviations

ALM: Appendicular lean mass
EWGSOP: European working group on sarcopenia in older people
HGS: Hand grip strength
Ht: Height
KES: Knee extension strength
LMM: Lower limb muscle mass
PFFS: Protein-rich food intake frequency score
SD: Standard deviation
SMI: Skeletal muscle index
Take10-FFS: Take 10 food frequency score
TUG: Timed up and go
UMM: Upper limb muscle mass
